# Energetic budget of diploid and triploid eastern oysters during a summer die-off

**DOI:** 10.3389/fmars.2023.1194296

**Published:** 2023-05-17

**Authors:** Sarah Bodenstein, Sandra M. Casas, Terrence R. Tiersch, Jerome F. La Peyre

**Affiliations:** 1 Aquatic Germplasm and Genetic Resources Center, School of Renewable Natural Resources, Louisiana State University Agricultural Center, Baton Rouge, LA, United States; 2 School of Animal Sciences, Louisiana State University Agricultural Center, Baton Rouge, LA, United States

**Keywords:** *Crassostrea virginica*, ploidy, cohorts, triploid mortality, summer mortality, physiology, scope for growth, dermo

## Abstract

Triploid oysters are widely used in off-bottom aquaculture of eastern oysters, *Crassostrea virginica*. However, farmers of the Gulf of Mexico (GoM) and Atlantic coast estuaries have observed unresolved, late-spring die-offs of triploid oysters, threatening the sustainability of triploid aquaculture. To investigate this, the physiological processes underlying oyster growth (e.g., feeding, respiration) and mortality of one-year-old diploid and triploid oysters were compared in early summer following an uptick in mortality. It was predicted that higher triploid mortality was the result of energetic imbalances (increased metabolic demands and decreased feeding behavior). Oyster clearance rates, percentage of time valves were open, absorption efficiency, oxygen consumption rates (basal and routine), ammonia excretion rate were measured in the laboratory and scope for growth was calculated. In addition, their condition index, gametogenic stage, *Perkinsus marinus* infection level, and mortality were measured. Mortality of triploids in the laboratory was greater than for diploids, mirroring mortality observed in a related field study. The physiological parameters measured, however, could not explain triploid mortality. Scope for growth, condition index, and clearance rates of triploids were greater than for diploids, suggesting sufficient energy reserves, while all other measurements where similar between the ploidies. It remains to be determined whether mortality could be caused from disruption of energy homeostasis at the cellular level.

## 1 Introduction

During the last decade, 2012–2022, off-bottom aquaculture has been promoted to supplement traditional on-bottom farming of eastern oysters (*Crassostrea virginica*) within U.S. Gulf of Mexico (GoM) estuaries (Walton et al., 2013). Off-bottom culture involves placing single-set, hatchery-produced seedstocks in some form of container that raises the oysters above the seafloor. This protects oysters from predation and sediment burial, allowing them to be grown in areas that would otherwise be unsuitable for oyster aquaculture (Moroney and Walker, 1999; Walton et al., 2013). Off-bottom farming, however, requires higher initial investments (i.e., in time, labor, and money) than on-bottom culture (Walton et al., 2013). To offset those costs triploid oysters (3N) are commonly grown because they can grow faster and be harvested throughout the summer months when diploid oysters (2N) have spawned and have poor meat quality (Allen Jr. and Downing, 1986; Walton et al., 2012). Triploid oysters have these advantages because of reduced or retarded gonadal development compared to diploid oysters (Allen Jr. and Downing, 1986; Dégremont et al., 2012). However, in recent years, oyster farmers of GoM and Atlantic USA estuaries have reported unexpected die-offs in late spring or early summer depending on the region, particularly of triploids (Guévélou et al., 2019; Wadsworth et al., 2019; Matt et al., 2020; Bodenstein et al., 2021).

The causes of these late-spring or early-summer die-offs often referred to as “triploid mortality events” in *C. virginica* remains unresolved. These events have not been associated with any single unfavorable environmental condition such as an abrupt change of temperature, salinity, pH, dissolved oxygen or sediment concentration nor any pathogen common to the U.S. GoM or mid-Atlantic regions (*Perkinsus marinus*, *Haplosporidium nelsoni*). The role of multiple environmental stressors in combination with reproductive stress and opportunistic infections as a cause of *C. virginica* triploid mortality, however, has not yet been investigated thoroughly. In some other oyster species, the causes of mortalities can be quite complex and take years to understand. In juvenile *Crassostrea gigas*, for example, worldwide mortalities referred to as “Pacific Oyster Mortality Syndrome” have been attributed to the interplay of a virus, *ostreid herpevirus* OsHV-1, and *Vibrio* bacteria while under the influence of various environmental and host factors (reviewed in Petton et al., 2021). The OsHV-1 virus initiates an infection, suppressing the oyster immune system and disrupting the microbiota, and the bacteria causes a secondary infection that eventually kills the oyster. Often, mortalities of oysters and other bivalves cannot be linked to any specific etiological agent (Burge et al., 2016; Go et al., 2017; King et al., 2019). In the case of *C. virginica*, neither *Perkinsus marinus* nor *Hapolosporidium nelsoni* load could explain the “triploid mortality events” reported in late-spring or early-summer, in recent studies (Guévélou et al., 2019; Wadsworth et al., 2019; Matt et al., 2020; Bodenstein et al., 2023). Moreover, histological observations of these triploid oysters indicated normal tissues with no obvious pathologies except for the gonads which showed reduced development and abnormal germ cells.

In *C. virginica*, the triploid die-offs are often accompanied by more limited mortalities of diploid oysters that occur when diploids have ripe gonads (e.g., advanced gametogenesis) and are ready to spawn or have spawned (Guévélou et al., 2019; Wadsworth et al., 2019; Matt et al., 2020). In GoM estuaries, diploid eastern mortality rates during this period of reproduction are generally low (<5% per month), unless also associated with long periods (>1 month) of low salinity (<5) or heavy *P. marinus* infection level (Casas et al., 2017; Wadsworth et al., 2019). In adult *C. gigas* diploid summer mortalities can be substantial and positively correlated with reproductive efforts (Koganezawa, 1974; Perdue et al., 1981; Samain et al., 2007; Cotter et al., 2010; Huvet et al., 2010; Jouaux et al., 2013). Moreover, Pacific oysters which only partially spawned and retained unspawned gametes displayed greater mortality than fully spawned oysters (Samain et al., 2007). It has been proposed that “metabolic disturbances” from the high energetic costs of developing gonadal tissues in oysters may divert energy resources away from basic cellular maintenance resulting in mortalities (Samain et al., 2007; Huvet et al., 2010). For example, during reproduction, when temperatures are elevated, *C. gigas* oyster immunity has been shown to be compromised resulting in a decrease ability to clear opportunistic pathogens (Li et al., 2012; Wendling and Wegner, 2013). Reproductive stress could also be a factor in the high triploid mortalities of *C. virginica* as in *C. gigas* (Houssin et al., 2019), considering the energetic cost of reproduction is greater in the earlier stages of gonad development (Honkoop, 2003) that seem to linger in triploids. Identifying physiological and metabolic changes accompanying triploidy may be useful in explaining differential mortalities of triploids relative to diploids during late spring and early summer.

Only one study has directly compared triploids and diploids in relation to some aspects of their energetic physiology (filtration, feeding and basal metabolism variables) (Mizuta et al., 2021). Measuring energy intake and expenditure to calculate scope for growth (i.e., the energy available for growth) of triploids as compared to diploids may yield insight as to what is causing C. virginica triploid mortality events. In June 2020, increased mortalities of one-year-old diploid and triploid eastern oysters produced at two GoM hatcheries were observed at a moderate-salinity (salinity of 12 – 26) field site and disproportionately affected triploids with no obvious causes (Bodenstein et al., 2023). To determine whether these differential mortalities were associated with differences in net energy balance between diploid and triploid oysters, we measured and compared clearance rates, percentage of time valves were open, absorption efficiencies, oxygen consumption rates, ammonia excretion rates and scopes for growth. We hypothesized that triploid mortality would be explained by greater metabolic demand (higher oxygen consumption and ammonia excretion rates), and decreased feeding behavior (lower clearance rates) or absorption efficiencies resulting in lower values of scope for growth.

## 2 Methods

### 2.1 Broodstock collection and spawning

In January 2019, about 300 wild broodstock oysters were collected from Sister Lake (SL; 29°14’45.0”N, 90°54’35.0”W), a public oyster seed ground in Louisiana with an annual salinity (mean ± SD) of 11.2 ± 5.5 [n = 10, 2009–2018] (USGS 07381349 water-quality monitoring station). These oysters were placed in longline bags for conditioning at the Louisiana Sea Grant Oyster Research Farm (LSURF) in Grand Isle, LA (23.3°C ± 6.3 and 19.0 ± 7.17 salinity from 2010–2020) (USGS #73802516, USGS, 2021). In June 2019, a portion of Sister Lake broodstock was spawned at the Louisiana Sea Grant Oyster Research Laboratory and Mike C. Voisin Oyster hatchery (LSURL). In July 2019, another portion was transported and spawned at Auburn University Shellfish Laboratory hatchery (AUSL) in Dauphin Island, Alabama.

At both hatcheries, diploids used in this study were produced by crossing wild female and male Sister Lake oysters. At LSURL 4 males and 3 females were mated and at AUSL, 33 males and 51 females were mated to produce F_1_ diploids. To produce the triploids used in this study, wild female Sister Lake oysters were crossed with male tetraploid oysters from one of two lines depending on the hatchery; 4DGNL17 line at the LSURL hatchery and 4MC18 line at the AUSL hatchery. The two tetraploid broodstock lines were originally part of the 4MGNL13 line, and each hatchery advanced the lines two generations to produce the two lines of tetraploids used in this study (Bodenstein et al., 2023). Sperm from tetraploid males in the 4DGNL17 and 4MC18 lines were verified by flow cytometry prior to fertilization of Sister Lake female broodstock. At LSURL 2 male tetraploids and 6 female diploids were mated and at AUSL, 8 male tetraploids and 51 female diploids were mated to produce F_1_ triploids. At LSURL, corresponding diploid and triploid crosses were not half-siblings, but at AUSL diploid and triploid crosses were half-siblings produced from the same females, using the same batches of eggs. For this study, F_1_ oysters produced in LSURL and AUSL will be referred to as LSU and AU cohorts. Diploid and triploid F_1_ oysters (crosses) produced in LSURL will be referred to as 2NLSU and 3NLSU. Diploid and triploid crosses produced at AUSL will be referred to as 2NAU and 3NAU.

Both hatcheries used standard spawning techniques (Wallace et al., 2008) and each cross was obtained as described previously (Bodenstein et al., 2023). After pediveliger larvae were set on microcultch substrate (~300 mm in diameter) to produce single-oyster spat, the spat were grown in an upwelling system from July to September at each hatchery until large enough to be deployed in the field (length ~11.0 – 16.4 mm). The AU cohort was transported to LSURL in September 2019 with authorization from the Louisiana Department of Wildlife and Fisheries, and oysters from each cohort were maintained in the longline system at LSURF. Ploidy verification by flow cytometry (Allen, 1983) was performed on oysters from both cohorts at LSURF in September 2019.

Following a significant increase in interval mortality rates of triploids (but not diploids) of the LSU cohort at LSURF from the end of April to mid-June (Table A1), oysters of both ploidies and cohorts (~210 of each) were transported in mid-June from the LSURF farm site to the Animal and Food Sciences Laboratory Building of the Louisiana State University Agricultural Center in Baton Rouge.

Oysters were scrubbed to remove any biofouling organisms (e.g., barnacles, algae), and placed in six 400-L tanks filled with aerated artificial seawater (Crystal Sea Marinemix, Marine Enterprises International, Baltimore, Maryland, USA) adjusted to a salinity of 15 ± 1 and water temperatures of 28 ± 1°C and equipped with biofilters. Each tank contained approximately 35 oysters from each cohort and ploidy. Oysters were fed ~5% of their fish Diet 1800^®^ once per day (Reed dry meat weight with Shell Mariculture, Campbell, CA).

After 10 d of acclimation to laboratory conditions, clearance rate (CR), valve opening, absorption efficiency (AE), and ammonia excretion rate (NR) were measured with a subset of oysters labeled for identification (Subset A, Table 1). At the same time, routine oxygen consumption rates (OCR) were measured with a different subset of oysters (Subset B Table 1). After routine OCR measurements were complete, basal OCR were measured on a third subset of oysters (Subset C, Table 1). Measurements were collected from individual oysters for all physiological rates except ammonia excretion, which was measured using three oysters in the same container. At the end of the study, oysters in Subset A were processed to determine shell height, gill area, dry meat weight, condition index as described below. Oysters in Subsets B and C were processed to determine dry meat weight. Finally, remaining oysters were used to determine *Perkinsus marinus* (dermo) infection level, and gametogenic stage (Subset D, Table 1). Mortality of all oysters was tracked every other day for the 6-week duration of the study and the percent cumulative mortality of oysters that died during the 6-week period was calculated (Ragone Calvo et al., 2003).

### 2.2 Clearance rates

Clearance rate (CR), defined as the volume of water cleared of suspended particles by an animal in a given amount of time, was measured using a static system (Riisgård, 1988; Casas et al., 2018a). Oysters were individually placed in 2-L beakers filled with gently aerated 0.5-mm filtered seawater with a salinity of 15. Oysters were fish Diet 1800^®^ was added left to acclimate for 1 h after which Shell to each beaker to bring the initial suspended particle concentration to 3 × 10^4^ cells mL^−1^. The per unit volume of algal particles ≥ than 5 mm was measured every 30 sec until particle counts declined below 50% of original values by use of a particle counter (PAMAS Model S4031 GO, PAMAS Partikelmess-und Analysesysteme, GMBH, Rutesheim, Germany). Beakers containing empty shells but the same concentration of algal particles were used as controls. Only beakers containing oysters with open valves were measured. The same oysters were used to measure clearance rate, absorption efficiency, and ammonia excretion rate.

Individual clearance rate $\left(CR_{i}\right)$ was calculated using the following equation:

$${CR}_{i}\left(L\;h^{-1}\right)=\left[\left(b-b^{'}\right)\times vol(L)\times 60{\;\min\;h}^{-1}\right.$$

where b is the slope of the linear regression between the natural logarithm of cell concentration (cells mL^−1^) and time (min) for the beaker with oyster, and b^’^ is the slope for the control beaker, vol is the volume of seawater in the beaker (2 L) and 60 (min h^−1^) is used to convert the time units. Clearance rates were standardized by shell height, specifically to a standard of 80 mm (i.e., average shell height for diploid and triploids, Casas et al., 2018b) using the equation:

$${CR}_{h}={\left(H_{\text{std}\;}/H_{\text{exp}\;}\right)}^{b}\times {CR}_{i}$$

where ${CR}_{h}$ is the clearance rate standardized by shell height, ${CR}_{i}$ is the individual oyster clearance rate, $H_{\text{std}}$ is the shell height of the standard oyster (80 mm), $H_{\text{exp}}$ is the shell height of the experimental oyster, and b is the allometric exponent, 1.78 (Cranford et al., 2011). Clearance rates were also expressed relative to gill area (CRa, L h^−1^ cm^−2^) to express the direct relationship between gill area and clearance rate (Meyhöfer, 1985; Riisgård, 1988) and to avoid standardization errors related to the different condition indices of the animals compared (Filgueira et al., 2008; Cranford et al., 2011). Finally, clearance rates were weight standardized to 1 g dry meat weight for the calculation of scope for growth according to the equation:

$${CR}_{W}={\left(W_{\text{std}\;}/W_{\text{exp}\;}\right)}^{b}\times {CR}_{i}$$

where ${CR}_{w}$ is the clearance rate standardized by dry meat weight, ${CR}_{i}$ is the rate of the experimental animal, $W_{\text{std}}$ is the standardization meat dry weight (1 g), $W_{exp}$ is the meat dry weight of the experimental oyster, and b is the allometric exponent, 0.58 (Cranford et al., 2011).

### 2.3 Valve opening

A non-invasive valvometry system (Casas et al., 2018b; Comeau et al., 2018), was used with 24 oysters (six oysters per cross) to estimate the percentage of time valves remained open during a 6-d period. A small magnet (5 × 3mm) was glued (using cyanoacrylate glue) to the left valve and a Hall element sensor (HW-300a, Asahi Kasei, Japan) coated in epoxy was glued directly across from the magnet on the right valve. The magnetic field in the form of output voltage (μV) was recorded at 1-min intervals by a dynamic strain recording device (DC 204R, Tokyo Sokki Kenkyujo Co., Shinagawaku, Tokyo, Japan). Oysters were continuously fed for one week at the supplier-recommended feeding rate of 0.05 ml of shellfish diet 1800^®^ (www.reedmariculture.com) per g of wet meat weight per day. Algae were added to a reservoir tank containing refrigerated water at salinity of 15, and food was dispensed continuously with a peristaltic pump to a 40-L tank holding the oysters. To allow for acclimation following the installation of sensors and magnets, the first day of valve monitoring was discarded from the dataset, and data analyses were restricted to days 2 to 7. Raw voltage data (voltage sec^−1^) were converted to display the average voltage each min. A regression equation was generated using the baseline (closed) voltage over the study period, to account for possible sensor drift over time. For each oyster, voltage was distributed in a 0 to 100% range, and oysters were considered closed when voltage was in the 0 to 10% range (Comeau et al., 2018). The number of “open” voltage data points was compared to the total number of voltage data points to calculate the percentage of time oyster valves remained open.

### 2.4 Absorption efficiency

Absorption efficiency (AE) was measured following the direct method in a static system (Smaal and Widdows, 1994). Oysters from each cross were individually placed in containers with 5 L of aerated 0.5-μm filtered water at salinity of 15. Containers held water but no oysters to serve as controls. Oysters were not fed the day before the assay and were cleaned (by scrubbing their shells) before placing them in the containers. Oysters were fed with 3 × 10^4^ cells mL^−1^ (a concentration at which no pseudofeces are produced) every 90 min for a total of 12 h and left undisturbed for 12 h before collection of feces. For each oyster, all feces produced during the 24-h experimental period were collected, as well as 750 ml of water at time 0 (TPM_0_) and 24 h (TPM_24_). Feces and water samples were filtered through pre-weighed filters (Whatman GF/C), rinsed with 0.5 M ammonium formate to eliminate salts, and dried for 24 h at 70°C to estimate feces dry weight (F) and food ingested dry weight (I), I=TPM_0_-TPM_24_. Absorption efficiency was calculated as:

AE=((I-F)/I)×100

### 2.5 Oxygen consumption rates

Oxygen consumption rates (OCR) of oysters fed daily (routine OCR) and oysters not fed for at least one-week (basal OCR) were measured in static 915-mL acrylic chambers filled with 0.5-μm filtered seawater with a salinity of 15 (Casas et al., 2018b). Chambers were sealed with clamps except for an opening into which a fitted self-stirring probe with optical dissolved oxygen sensors (ProOBOD, YSI Incorporated, Yellow Springs, OH, USA) was inserted. Dissolved oxygen readings began after an oyster opened its valves and continued until the dissolved oxygen level fell to 70% of the starting level (Casas et al., 2018b). Chambers containing empty shells were used as controls. Individual oyster oxygen consumption rates $\left({OCR}_{i}\right)$, basal and routine, were calculated using the equation:

$$\left.{OCR}_{i}=([(b-b\})\times Vol]\times 60\;min{\;h}^{-1}\right)$$

where b is the slope of the linear regression of oxygen concentration (mg L^−1^) in the chamber *vs*. time, b^’^ is the slope for the control, Vol is the volume of the chamber minus oyster volume. Oxygen consumption rates were standardized to 1 g dry meat weight following the formula structure described above for CRw and a weight exponent, b, of 0.58 (Casas et al., 2018b).

$${OCR}_{W}={\left(W_{\text{std}\;}/W_{\text{exp}\;}\right)}^{b}\times {OCR}_{i}$$

### 2.6 Ammonia excretion rate

The rate of ammonia excretion (NR) was determined by placing three oysters of each cross in 1-L beakers filled with 1 L of 0.5-μm filtered seawater with a salinity of 15 (Widdows and Johnson, 1988; Kinsella, 2019). A beaker containing 0.3 L of seawater was used as a control. After 4 h, samples of water were collected to measure ammonia levels using an ammonia ion electrode (Model Truline, YSI Incorporated, Yellow Springs, OH, USA). Ammonia excretion rates were measured six times for each cross (2NAU, 3NAU, 2NLSU, 3NLSU) using different oysters each time. Six controls using empty shells were also measured for a total of 30 measurements.

Ammonia excretion rate was expressed relative to meat dry weight (mg NH_3_ h^−1^ g^−1^). The NR was calculated using the equation:

$$NR\;=\;([(n\;-\;n^{\prime })\;\times \;Vol]/time)/g$$

where n is the ammonia level for the beaker with the oysters (μg/L), n’ is the ammonia level for the control beaker (μg/L), Vol is the volume of the beaker (0.3 L), h is the amount of incubation time (4 h), and g is the combined dry weight of the oysters in the beaker in grams.

### 2.7 Scope for growth

Scope for growth (SFG) is an index of energy available for growth and reproduction. Scope for growth is estimated from the difference between energy absorbed from the food and the energy expended *via* respiration and excretion (Widdows, 1985; Smaal and Widdows, 1994). The SFG was calculated by using the equation:

SFG=A-(R+U)

where A is the energy absorbed, R is the energy respired and U is the energy excreted (Widdows and Johnson, 1988; Domínguez et al., 2020; Montagnac et al., 2020).

To obtain A, the energy consumed (C,J/h) was first calculated using the equation:

$$C\;=\;CRw\;\times \;open\;\%\;\times \;POM\;\times \;23:5J\;{mg}^{-1}\;POM$$

where CRw are the clearance rate values (L h^−1^ g^−1^) for oysters (Subset A) in each cross, open% is the percent time valves were open, POM is the particulate organic matter (mg L^−1^), and 23.5 J mg^−1^ POM is the Joules in 1 mg of POM (Bayne and Newell, 1983). Energy consumed values were calculated for individual oysters in each cross. Individual energy consumed values were multiplied by the average absorption efficiency (AE) for each cross to obtain A values for oysters in each cross. The energy absorbed (A) values were averaged to obtain one value per cross.

The routine OCR values for oysters (Subset B) in each cross (mg O_2_ h^−1^ g^−1^) were multiplied by the number of Joules needed to respire 1 mg of O_2_ (14.06 J mg^−1^ O_2_) (Gnaiger, 1983) to calculate the energy respired, R. The energy respired values for individual oysters in each cross were then averaged to obtain one value per cross.

Finally, the average energy excreted for each cross, U, was calculated by multiplying the average ammonia excretion rate for each cross (mg NH_3_ g^−1^ h^−1^) by the number of Joules needed to excrete 1 mg of NH_4_ (25.1 J mg^−1^ NH_4_) (Gnaiger, 1983, Bayne, 2017). Therefore, one energy excreted value was calculated for each cross. The average energy absorbed, respired, and excreted values for each cross were used in the SFG equation to calculate a single average SFG for each cross (2NAU, 3NAU, 2NLSU, 2NLSU).

### 2.8 Condition index, *P. marinus* infection level, and gametogenic stage

After completing all physiological measurements, oyster shell height, and whole oyster weight and volume were determined. Oysters were opened and an image of the gill was captured by digital camera (Nikon Coolpix S9600, Tokyo, Japan) (Casas et al., 2018a). Gill area (cm^2^) of all 8 lamella, as seen from a top-down view, was estimated using image analysis software (ImageJ, version 1.53a, National Institutes of Health, USA) that was used to calculate clearance rate by gill area. Additionally, a linear regression analysis was performed to examine the effect of ploidy and shell height on gill area.

Oyster meat from oysters in Subsets A, B, and C were dried at 70° C for 48 h to determine the meat dry weight used to standardize the physiological rates (CR, routine OCR, basal OCR, and NR). Dry meat weights from oysters in Subset A were also used to calculate condition index (CI). The ratio of body mass to cavity shell volume (CI) was calculated using the equation:

CI=(meatdrywt/(wholeoysterwt-wetshellwt))×100

(Abbe and Albright, 2003).

Additionally, at the end of the study, 15 oysters from each cross were collected and processed to analyze *P. marinus* infection level using the whole-oyster procedure (La Peyre et al., 2018). Eight oysters from each cross were cross-sectioned and processed by standard histological technique (Howard et al., 2004) to determine sex and gametogenic stage. The stages of gametogenic development were distinguished based on morphology of the follicles, follicle contents, and a visual estimate of the percentage of the incipient gonad area, the area between the digestive tissue and mantle, occupied by gonadal follicles, or follicle coverage as described by Matt and Allen (2021). These stages were inactive (≤ 5% follicle coverage), very early active (10–30% follicle coverage), early active (follicles with lumina and 10–40% follicle coverage), active (50–70% follicle coverage), late active (pronounced follicle canals with 75–90% follicle coverage), ripe (follicles filled with oocytes or spermatozoa and ≥ 80% follicle coverage), spawning (slight follicle contraction with 60–90% follicle coverage), advanced spawning (greater follicle contraction with 30–70% follicle coverage), and spawned out (collapsed follicles with ≤ 50% follicle coverage) (Matt and Allen, 2021).

### 2.9 Data analysis

All statistical analyses were performed in RStudio (version 4.0.3, R Core Team, 2020). Clearance rate, gill area, condition index, percentage of time valves were opened, absorption efficiency, oxygen consumption rate, and ammonia excretion rate were examined for normality (Shapiro-Wilk) and homogeneity of variance (Bartlett test), transformed as required and analyzed with a two-factor (i.e., cohort and ploidy) analysis of variance (ANOVA) followed by *post-hoc* Tukey-Kramer pair-wise comparisons when significant differences were found (p< 0.05). A linear regression model was used to analyze the effect of shell height and ploidy on gill area. A linear regression model was used to analyze the effect of ploidy and cohort on infection level. A linear mixed effect model (R package nlme) was used to analyze the effects of cohort and ploidy (fixed effects), and tank (random effect) on cumulative mortality.

## 3 Results

### 3.1 Morphology, clearance rates, and valve opening

**FIGURE 1:**
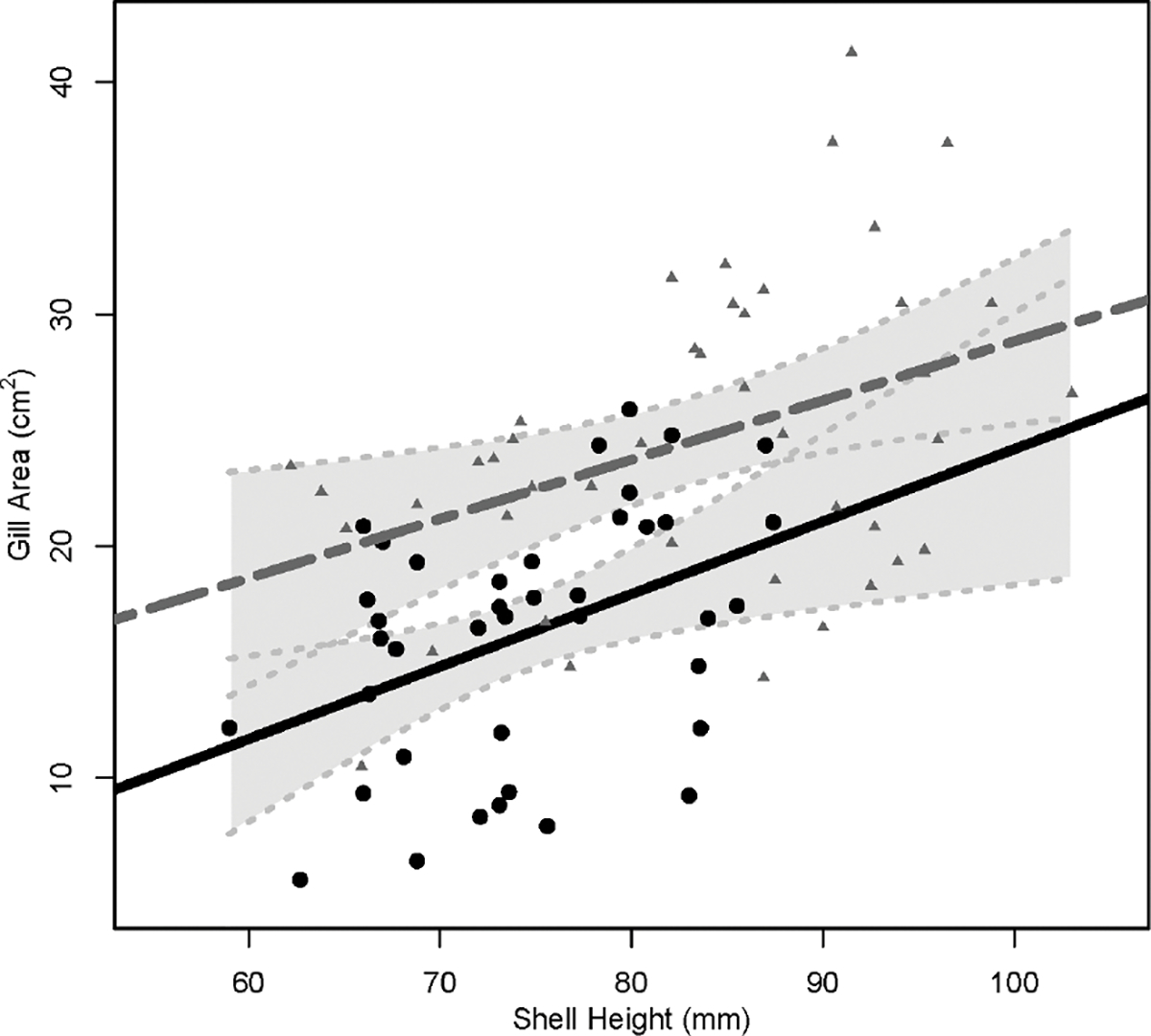
Linear regression lines for the relationship of shell height (mm) and gill area (cm2 ) in diploids (solid, black line) and triploids (dashed, gray line). Circles represent shell heights and gill areas of individual diploids and gray triangles represent shell heights and gill areas of individual triploids. Light gray, shaded areas behind regression lines represent 95% confidence intervals, with light gray dotted lines indicating upper and lower 95% confidence limits.

Triploids were longer than diploids (*P*< 0.001, 3N: 83.4 ± 9.9 mm, 2N: 74.6 ± 7.3 mm), and no differences in shell height were found between cohorts (*P* = 0.19, LSU: 77.8mm, AU: 80.3 mm). Triploids also had larger gill areas (24.62 ± 6.56 cm^2^) than diploids (16.22 ± 5.37 cm^2^) across cohort (*P*< 0.001) (Table 2). When the relationship between shell height and gill area was compared between ploidies, triploids with the same shell heights as diploids had larger gill areas, *P* ≤ 0.001 (Figure 1).

The larger triploids had greater individual clearance rates, 5.15 ± 3.46 L h^−1^, than diploids, 2.78 ± 2.11 L h^−1^ (*P*< 0.001). Furthermore, when clearance rates standardized by shell height (CRh) were compared, triploids had greater (*P* = 0.01) clearance rates (5.01 ± 3.23 L h^−1^ 80 mm^−1)^ than diploids (3.24 ± 2.53 L h^−1^ 80 mm^−1^), with no differences found between cohorts (Table A3, LSU: 4.14, AU: 4.22). When clearance rates standardized by gill area (CRa) were compared, oysters from both cohorts and ploidies had similar clearance rates, 0.21 ± 0.16 L h^−1^ cm^−1^ (*P* ≥ 0.23, for all comparisons) (Table 2). Finally, oyster valve movement across cohort and ploidy was not different (*P* ≥ 0.25, for all cases). Oysters opened their valves 59% ± 5 of the time on average.

### 3.2 Condition index

Triploids had higher condition indices (6.0 ± 1.64) than diploids (3.1 ± 0.9) (P ≤ 0.01, for all comparisons).

### 3.3 Absorption efficiency

The absorption efficiencies (72% ± 18 on average) were not different between ploidies or between cohorts, (P ≥ 0.31, for all comparisons) (Table 2).

### 3.4 Oxygen consumption rates

The average basal OCR_w_ of all crosses (1.48 ± 0.50 mg O_2_ h^−1^ g^−1^) were reduced by 32% compared with routine OCR_w_ (2.19 ± 0.66 mg O_2_ h^−1^ g^−1^). Oysters across cohort and ploidy had similar routine OCR_w_ (P ≥ 0.05, for all cases) (Table 3). However, basal OCR_w_ were affected by cohort (P ≤ 0.001). Oysters of the LSU cohort had lower basal OCR_w_ (1.32 ± 0.48 mg O_2_ h^−1^ g^−1^) than oysters of the AU cohort, 1.65 ± 0.48 mg O_2_ h^−1^ g^−1^. Differences in basal OCR_w_ between triploid (1.52 ± 0.51 mg O_2_ h^−1^ g^−1^) and diploid (1.44 ± 0.50 mg O_2_ h^−1^ g^−1^) oysters were not observed (P = 0.56).

### 3.5 Ammonia excretion rate

The average ammonia excretion rate (NR) for all crosses was 12.65 ± 17.17 g NH_3_ h^−1^ g^−1^. Ammonia excretion rate was only affected by cohort (P = 0.04). Oysters in the LSU cohort had lower NR (8.26 ± 12.17 g NH_3_ h^−1^ g^−1^) than did oysters in the AU cohort (17.04 ± 20.64 g NH_3_ h^−1^ g^−1^. No differences in NR between diploid (15.14 ± 17.71 g NH_3_ h^−1^ g^−1^) and triploid (10.17 ± 15.94 g NH_3_ h^−1^ g^−1^) oysters were found (Table 3).

### 3.6 Scope for growth

Diploid oysters in the LSU and AU cohorts had the lowest average SFG values (Table 4). Triploid oysters (of both cohorts) had higher average SFG values than either diploid cohort, and triploids in the LSU cohort had the highest average SFG value (Table 4). Triploid oysters also had higher energy absorption values (21% higher on average) and lower energy excreted (39% lower on average) than diploids (Table 4).

### 3.7 *Perkinsus marinus* infection level

Triploids had slightly higher infection intensities (3.79 ± 1.72 log_10_ parasites g^−1^ wet tissue), on average than diploids (3.69 ± 1.62 log_10_ parasites g^−1^ wet tissue) (general linear regression, t = 2.30, P = 0.03). Most oysters sampled (63%) had light infection intensities (< 10^4^ parasites g^−1^ wet tissue), while 20% had moderate (10^4^ - 5 × 10^5^ parasite g^−1^ wet tissues) and 17% had heavy (> 10^5^ parasite g^−1^ wet tissues) infection intensities (Table 4, Casas et al., 2017). No difference was found between the infection intensities of oysters in the LSU cohort (3.82 ± 1.62 log_10_ parasites g^−1^ wet tissue) and the AU cohort (3.66 ± 1.71 log_10_ parasites g^−1^ wet tissue, P = 0.06, Table A2).

### 3.8 Gametogenic stage

Of all oysters sampled at the end of the study that had an active or spawning gonadal stage, 64% were males and 36% were female. Most of the triploids were in the inactive gonadal stage (≤ 5% follicle coverage, LSU: 63%, AU: 88%) or in post-spawning stage with collapsed follicles, hemocytes invading the gonad, and few residual gametes (LSU: 25%, AU: 12%). Only one triploid oyster was observed in advanced spawning stage (LSU: 12%, AU: 0%). Among diploids, most were in the inactive gonadal stage (LSU: 25%, AU: 25%) or spawned out (LSU: 62%, AU: 38%), but there were oysters in spawning stage in the Auburn cohort (25%) and advanced spawning stage in both cohorts (LSU:12%, AU:12%).

### 3.9 Cumulative mortality

In the laboratory, triploids experienced higher percent mortality (8.9 – 12.1% range) than diploids (2.5 – 2.9% range) over the experiment (linear mixed effects model, t = 3.33, P< 0.001). However, the percent mortality of the LSU cohort (8 ± 7% average) was not different from that of the AU cohort (6 ± 7% average) (linear mixed effects model, t =0.74, P = 0.47).

## 4 Discussion

The goal of this study was to evaluate if differences in the energetic physiology of triploids and diploids could explain an observed late spring “triploid mortality” event. Six main physiological parameters were measured to determine feeding behavior and metabolism: clearance rate (CR), valve movement, absorption efficiency (AE), basal and routine oxygen consumption rate (OCR), and ammonia excretion rate (NR). These parameters are related, as when oysters open their valves they feed, perform aerobic respiration, and excrete waste (Bayne, 2017; Casas et al., 2018b). The physiological parameters were used to calculate scope for growth (SFG), a function of energy absorbed minus the sum of energy respired and energy excreted. We hypothesized that triploid mortality observed in the field would be explained by increased metabolic demands, decreased feeding behavior, and lower SFG values.

Contrary to the hypothesis of this study, higher triploid mortality could not be explained by the measured physiological parameters. All SFG values fell within the range of SFG values reported for temperate and tropical bivalves (−10.1 – 89.4 J h^−1^ g^−1^, Domínguez et al., 2020; 3.7 – 72.6 J h^−1^ g^−1^ of dry weight, Kesarcodi-Watson et al., 2001). The LSU triploids had SFG values classified as having high growth potential (> 15 J g^−1^ h^−1^, Widdows et al., 2002). Triploid Auburn oysters had SFG values that fell into the moderate growth potential category (5 – 15 J g^−1^ h^−1^, Widdows et al., 2002). Auburn and LSU diploids had SFG values classified as low growth potential (< 5 J g^−1^ h^−1^, Widdows et al., 2002). A low SFG value would indicate that the energy expended was similar to energy absorbed, and that the animal was in stressful conditions. No associations between low SFG values and high mortality were found in this study. Moreover, despite higher mortality, triploid oysters not only had higher SFG but the condition index values, which indicated how much of the shell cavity was occupied by tissue and a reflection of potential energy reserve, were also greater than diploids.

The higher SFG of triploid oysters was in line with the expectation of greater growth rate of triploid oysters observed in the field (Allen Jr. and Downing, 1986; Dégremont et al., 2012). Scope for growth values represent the net energy that should be available for processes such as somatic growth and meeting metabolic demands (Bayne and Newell, 1983). In accordance with their higher SFG values, triploids had faster growth rates in the field compared to diploids (Bodenstein et al., 2023). Triploids had higher growth rates than diploids because triploids had higher clearance rates when compared at individual basis (CRi) or when standardized by shell height (CRh). This was likely due to the larger average gill area per mm of shell height of triploids (Figure 1). As gill area of a bivalve increases so does the capacity to filter suspended particles from the water column, i.e., pumping, filtration, or clearance rates (Meyhöfer, 1985; Riisgård, 1988; Jones et al., 1992). Therefore, triploids consumed a greater amount of energy which in turn led to greater energy absorbed and finally greater SFG values.

It was predicted that triploid oysters, with their higher growth rates, would have higher oxygen consumption rates (metabolic demands) than diploid oysters. Higher oxygen consumption rates can lead to an imbalance between production and elimination of reactive oxygen species (ROS), resulting in a lethal oxidative stress (Buttemer et al., 2010). Triploids have been observed to have higher rates of digestive enzyme activity and increased ROS production, when compared to diploids (*C. gigas*, Haberkorn et al., 2010). These observations led to the hypothesis that triploids have higher metabolic activity than diploids (Haberkorn et al., 2010) and these higher metabolic demands could be the cause of increased triploid mortality. Moreover, developing and maintaining gonadal tissues over an extended period at high temperatures, without spawning in triploids could elevate metabolism and contribute to oxidative stress and eventually death (Lesser, 2006). Higher respiration rates have been observed in oysters with higher investment in gametogenesis (Bayne and Widdows, 1978; Casas et al., 2018b). In Pacific oysters (*C. gigas*), higher respiration rates and ROS levels were reported before mortality events (Samain, 2011). Higher energy expenditure has long been observed to be predictive of natural mortality with the oxidative stress theory being the most generally accepted explanation (Speakman et al., 2002; Hulbert et al., 2007). Our results, however, showed that this was not the case in our study because no difference could be shown between the oxygen consumption rates of triploid and diploid oysters. Finally, the other parameters (valve movement, clearance rate standardized by gill area, and absorption efficiency) were all similar between the ploidies meaning that feeding behavior, gill functioning or digestive efficiency could not be found to explain triploid mortality.

The physiological parameters analyzed in this study attempted to measure energy balance at the organismal level to explain the differential die-offs in late spring and early summer between diploid and triploid oysters. Energy balance could have been disrupted on a smaller scale, such as at the cellular or chromosomal levels (Sokolova et al., 2012; Newman and Gregory, 2019). Looking first at the cellular level, triploid cells have larger cell volumes than diploid cells due to increased genomic content. This would result in increased intracellular distances and reduced surface-area-to-volume ratios (Hinegardner, 1974; Guo and Allen, 1994; Miettinen et al., 2017). Transporting molecules over greater distances within a cell can reduce the rate of biochemical reactions. Limiting the surface area of a cell in relation to volume slows down nutrient and oxygen uptake as well as waste disposal on the cellular level. These factors can lead to localized metabolic inefficiency and cell death which may not be detected at the organismal level, but that may eventually lead to organismal death (Miettinen et al., 2017).

Looking at another scale, cells within triploid animals could have had chromosomal abnormalities such as aneuploidy, a condition in which the chromosome number is not an exact multiple of the haploid number (Wachtel and Tiersch, 1993). Triploid oysters have been observed to contain a higher proportion of aneuploid cells than diploids (C*. virginica*, de Sousa et al., 2016; *G. gigas*, Wang et al., 1999) and negative correlations between individual level of aneuploidy, growth, and survival have been observed in bivalves (*Pinctada fucata*, Komaru and Wada, 1994; *C. gigas*, Leitão et al., 2001). Moreover, chromosome alterations (loss or addition) can be extensive during cell division in triploid oysters (de Sousa et al., 2016; Wang et al., 1999), altering the relative expression of hundreds of genes, compromising cellular functions, and causing cell death (Sheltzer and Amon, 2011; Vitale et al., 2011; Storchova, 2012; Rutledge and Cimini, 2016). These events would be expected to be most pronounced at a time when cells are actively dividing and differentiating, such as during larval growth, in early life stages, or during gametogenesis, at a later life stage. Anecdotally, higher mortality of triploids compared to diploids are observed in hatcheries during larval development stages (Dr. Brian Callam, Mike Voisin Oyster Hatchery, LSU, pers. comm.). It is possible excessive cell death from chromosome alterations (Storchova, 2012) may overwhelm the immune responses of triploids and increase their susceptibility to opportunistic pathogens (Van De Peer et al., 2021). On the other hand, the circulating immune cells (hemocytes) of triploid oysters have been reported to be larger, produced more reactive oxygen species which have antimicrobial activity, were capable of ingesting a higher number of foreign particles and had a significantly greater capacity to kill *Vibrio* species suggesting a more capable immune function that would increase resistance to pathogens (La Peyre et al., 1999; Gagnaire et al., 2006; Haberkorn et al., 2010). Future studies can operate at multiple levels and evaluate physiological parameters (organismal level), cell size and intracellular metabolite gradients (cellular and mitochondrial level), and karyotype (chromosomal level) to get a more complete view of how these levels interact and influence oyster bioenergetics.

Finally, triploids and diploids used in this study comprised animals from two cohorts produced at two hatcheries, adding another layer of complexity to analyzing the causes of triploid mortality. Cohort differences may have been caused by hatcheries personnel using different numbers of parents during spawning. A greater number of parents (both male and female broodstock) was used at AUSL than at LSURL when producing F_1_ triploids and diploids (Table 1 in Bodenstein et al., 2023). Therefore, Auburn oysters could have had higher heterozygosity and genetic diversity (Aho et al., 2006; Hughes et al., 2019) which has been associated with increased survival in bivalves (*Ruditapes decussatus*, Borsa et al., 1992; *C. virginica*, Zouros and Foltz, 1983). Organisms with higher genetic diversity and phenotypic plasticity are often better able to survive sudden environmental changes, providing time for acclimation to occur (Li et al., 2021; Sirovy et al., 2021). It has been suggested that phenotypic plasticity is particularly important for sessile organisms located in estuarine environments because they cannot migrate to avoid fluctuations in environmental conditions (Li et al., 2021). Another factor that has been associated with decreased heterozygosity is inbreeding depression. As proposed in Bodenstein et al. (2023), inbreeding of tetraploid lines could have occurred because of the generally high genetic load of oysters (Plough, 2016). In a study with Pacific oysters, the cause of inbreeding depression was determined to be selection against deleterious, recessive mutations of which oysters have been observed to carry a large number (minimum of 8 – 14 per genome, Launey and Hedgecock, 2001). The selection against recessive mutations and resulting inbreeding of tetraploid parents that contribute a greater amount of genetic material than diploid parents, could have reduced the heterozygosity of triploid offspring affecting their survival.

## 5 Conclusions

Triploid mortality could not be explained with the energetic physiology measurements taken in this study. In contrast to predictions, triploids did not have higher metabolic demands than diploids, and triploids had higher SFG values than diploids. It is important to note that our measurements were made in the laboratory with oysters maintained under constant environmental conditions. Oysters in estuaries, in contrast, are exposed to continuously changing and sometimes extreme environmental conditions which they must cope with as osmoconformers and poikilotherms. In future experiments physiological parameters, such as clearance and respiration rates and absorption efficiency, should be measured following exposure of oysters to a combination of stressors in the laboratory or *in situ* to more accurately define the effect of multiple or fluctuating environmental conditions on the physiology and mortality of diploid and triploid oysters (Pouvreau et al., 2000; Grizzle et al., 2008; Gray and Langdon, 2018).

## Supplementary Material

Supplementary table

## Figures and Tables

**TABLE 1:** The number of oysters per cross (2NAU, 3NAU, 2NLSU, 3NLSU) and the subset of oysters that were sampled for each physiological measurement.

Subset	Physiological Measurement	Cross	Number of Oysters per Cross
A	Shell Height	All	24
	Gill Area	All	24
	Dry Meat Weight	All	24
	Condition Index	All	23
	Clearance Rate	2N AU	18
		3N AU	21
		2N LSU	19
		3N LSU	21
	Absorption Efficiency	2N AU	30
		3N AU	28
		2N LSU	22
		3N LSU	27
	Ammonia Excretion	All	18
	% time Valves are Open	All	6
B	Routine Oxygen Consumption Rate & Dry Meat Weight	2N AU	23
		3N AU	24
		2N LSU	24
		3N LSU	24
C	Basal Oxygen Consumption Rate & Dry Meat Weight	2N AU	18
		3N AU	21
		2N LSU	20
		3N LSU	19
D	*P. marinus* infection level	All	15
	Gametogenic Stage	All	8

The same oysters within a subset were used for all measurements listed within that subset. If “All” is listed the same number of oysters per cross were sampled for that measurement.

**TABLE 2:** Mean ± standard deviations for shell height, gill area, individaul clearance rate (CRi), clearance rate standardized by height (CRh), clearance rate standardized by gill area (CRa), clearance rate standardized by weight (CRw), percentage of time valves were open, and percent absorption efficiency (AE) for each oyster cross (2NAU, 3NAU, 2NSLU, 3NSLU).

Ploidy	Cohort	Shell Height (mm)	Gill Area (cm^2^)	CRi (L h^−1^)	CRh (L h^−1^ 80 mm^−1^)	CRa (L h^−1^ cm^−2^)	CRw (L h^−1^ g^−1^)	% Valve Open	AE (%)
2N	AU	75.6 ± 7.48	17.42 ± 5.91	3.23 ± 2.10	3.75 ± 2.64	0.19 ± 0.13	4.60 ± 2.70	57.52 ± 8.28	71.8 ± 19.3
2N	LSU	73.7 ± 7.06	15.12 ± 4.72	2.35 ± 2.07	2.76 ± 2.38	0.18 ± 0.19	3.86 ± 3.89	53.72 ± 12.82	74.9 ± 17.3
3N	AU	84.1 ± 10.9	25.45 ± 6.79	4.87 ± 3.12	4.62 ± 2.88	0.22 ±0.16	4.62 ± 3.16	64.53 ± 9.85	67.1 ± 22.9
3N	LSU	81.5 ± 9.45	23.82 ± 6.37	5.43 ± 3.83	5.39 ± 3.57	0.24 ±0.15	5.00 ± 3.32	62.10 ± 20.08	72.6 ± 12.4

**TABLE 3:** Mean ± standard deviations for basal and routine oxygen consumption rates (basal and routine OCR_w_), and ammonia excretion rate (NR) for each oyster cross (2NAU, 3NAU, 2NLSU, 3NLSU).

Ploidy	Cohort	basal OCR_w_ (mg O_2_ h^−1^ g^−1^)	routine OCR_w_ (mg O_2_ h^−1^ g^−1^)	NR (*μ*g NH_3_ h^−1^ g^−1^)
2N	AU	1.49 ± 0.43	2.49 ± 0.90	21.9 ± 20.7
2N	LSU	1.39 ± 0.56	2.05 ± 0.50	8.39 ± 15.1
3N	AU	1.77 ± 0.49	2.15 ± 0.46	12.2 ± 21.2
3N	LSU	1.25 ± 0.37	2.06 ± 0.62	8.13 ± 9.88

**TABLE 4:** Average values for P. marinus infection level, condition index, cumulative mortality, energy absorbed (A, J h^−1^ g^−1^), energy respired (R, J h^−1^ g^−1^), energy excreted (U, J h^−1^ g^−1^), and Scope for Growth (SFG, J h^−1^ g^−1^) for each oyster cross (2NAU, 3NAU, 2NLSU, 3NLSU).

Ploidy	Cohort	*P. marinus* level (log_10_ cell g^−1^)	Condition Index	Cumulative Mortality (%)	A (J h^−1^ g^−1^)	R (J h^−1^ g^−1^)	U (J h^−1^ g^−1^)	SFG (J h^−1^ g^−1^)
2N	AU	3.04 ± 1.35	3.13 ± 1.13	2.48 ± 2.93	40.19	35.01	0.55	4.63
2N	LSU	4.40 ± 1.58	3.13 ± 0.54	2.94 ± 4.56	32.84	28.82	0.21	3.81
3N	AU	4.00 ± 1.67	5.77 ± 1.50	8.89 ± 8.45	42.30	30.23	0.31	11.77
3N	LSU	3.62 ± 1.60	6.19 ± 1.80	12.10 ± 5.91	47.65	28.96	0.20	18.49

Only one value was calculated per cross for A, R, U, and SFG, therefore no standard deviation values are provided.

## Data Availability

The raw data supporting the conclusions of this article will be made available by the authors, without undue reservation.
