# Supplementary material for: Energetic budget of diploid and triploid eastern oysters during a summer die-off
Source: Front Mar Sci. Author manuscript; Available in PMC 2024 Apr 4. (PMC10993659; doi:10.3389/fmars.2023.1194296)
Supplement: Supplementary table [file NIHMS1960282-supplement-Supplementary_table.pdf]

Supplementary Material

Table A1. The average interval mortality per day and shell height (± standard deviation) for four sampling intervals at the LSURF field site or in the laboratory for diploid and triploids oysters (2N and 3N) of both cohorts (AU and LSU) spawned in June 2019 using Sister Lake broodstock.

Sampling Interval	Temperature (°C)	Salinity	Ploidy	Cohort	Interval Mortality (%)	Mortality rate (%/d^{-1})	Shell Height (mm)
3/13/20-4/20/20 Field	23.3 ± 1.7	10.1 ± 3.7	2N	AU	0.0	0.00	59.5 ± 3.9
			3N	AU	1.0	0.02	68.5 ± 3.1
			2N	LSU	0.3	0.01	63.5 ± 3.3
			3N	LSU	2.0	0.05	71.0 ± 3.0
4/20/20 - 6/18/20 Field	23.8 ± 5.9	12.1 ± 7.6	2N	AU	1.0	0.02	69.7 ± 3.5
			3N	AU	1.0	0.02	78.5 ± 2.0
			2N	LSU	0.6	0.01	71.6 ± 3.5
			3N	LSU	18.7	0.31	78.5 ± 3.7
6/18/20 - 7/14/20 Field	29.5 ± 1.7	11.8 ± 3.8	2N	AU	10.0	0.37	73.9 ± 3.2
			3N	AU	10.7	0.40	83.7 ± 2.4
			2N	LSU	10.5	0.39	76.1 ± 2.8
			3N	LSU	13.2	0.49	81.5 ± 2.8
7/24/20-9/9/20 Laboratory	27 ± 1	15 ± 1	2N	AU	2.5	0.05	75.7 ± 7.5
			3N	AU	8.9	0.19	84.2 ± 10.9
			2N	LSU	2.9	0.06	73.7 ± 7.1
			3N	LSU	12.1	0.26	81.5 ± 9.4

Table A2. Results of linear regression model for the effects of cohort and ploidy on P. marinus infection intensity, and results of linear mixed effects model for the effects of cohort and ploidy on cumulative mortality.

Dependent Variable	Independent Variable	DF	Estimate Std.	Std. Error	t value	P value
Infection Intensity	Intercept	56	2.84	0.43	6.56	**<0.01**
	cohort	56	1.15	0.60	1.91	0.06
	ploidy	56	1.26	0.61	2.06	**0.04**
	cohort:ploidy	56	-1.64	0.87	-1.89	0.06
Mortality	Intercept	39	2.00	2.07	0.96	0.34
	cohort	39	1.22	2.04	0.60	0.55
	ploidy	39	6.09	2.04	2.98	**<0.01**
	cohort:ploidy	39	4.76	2.89	1.65	0.11

Table A3. Results of two-factor ANOVA analyses used to test the effects of cohort and ploidy on shell height, gill area, clearance rate measurement (CRi, CRh, CRa, CRw), condition index (CI), percentage time open, absorption efficiency (AE), basal and routine oxygen consumption rate (OCRw), and ammonia excretion rate (NR).

Dependent Variable	Independent Variable	Df	Sum Sq	Mean Sq	F value	P value
Shell Height	cohort	1	141.00	141.00	1.75	0.19
	ploidy	1	1488.00	1488.40	18.51	**<0.01**
	cohort:ploidy	1	3.00	2.80	0.04	0.85
Gill Area	cohort	1	110.50	110.50	3.05	0.08
	ploidy	1	1445.50	1445.50	39.89	**<0.01**
	cohort:ploidy	1	3.00	3.00	0.08	0.77
CRi	cohort	1	0.01	0.01	0.03	0.86
	ploidy	1	3.97	3.97	15.54	**<0.01**
	cohort:ploidy	1	0.94	0.94	3.66	0.06
CRh	cohort	1	0.29	0.29	0.33	0.57
	ploidy	1	6.00	6.00	6.85	**0.01**
	cohort:ploidy	1	1.72	1.72	1.96	0.16
CRa	cohort	1	0.02	0.02	0.05	0.82
	ploidy	1	0.55	0.55	1.48	0.23
	cohort:ploidy	1	0.23	0.23	0.63	0.43
CRw	cohort	1	0.12	0.12	0.34	0.56
	ploidy	1	0.27	0.27	0.74	0.39
	cohort:ploidy	1	0.51	0.51	1.42	0.24
CI	cohort	1	0.25	0.25	0.14	0.71
	ploidy	1	129.52	129.52	73.98	**<0.01**
	cohort:ploidy	1	0.68	0.68	0.39	0.54
Time Open	cohort	1	42.19	42.19	0.24	0.63
	ploidy	1	258.41	258.41	1.45	0.25
	cohort:ploidy	1	1.97	1.97	0.01	0.92
AE	cohort	1	187862.00	187862.00	0.15	0.70
	ploidy	1	1320523.00	1320523.00	1.05	0.31
	cohort:ploidy	1	288402.00	288402.00	0.23	0.63
Basal OCRw	cohort	1	2.02	2.02	8.97	**<0.01**
	ploidy	1	0.08	0.08	0.36	0.55
	cohort:ploidy	1	0.84	0.84	3.75	0.06
Routine OCRw	cohort	1	0.14	0.14	3.93	0.05
	ploidy	1	0.05	0.05	1.37	0.24
	cohort:ploidy	1	0.04	0.04	1.15	0.29
NR	cohort	1	1331.70	1331.70	5.41	**0.04**
	ploidy	1	18.20	18.20	0.07	0.79
	cohort:ploidy	1	574.00	574.00	2.33	0.15
